# Ex Situ Stabilization/Solidification Approaches of Marine Sediments Using Green Cement Admixtures

**DOI:** 10.3390/ma17143597

**Published:** 2024-07-21

**Authors:** Pravendra Yadav, Andrea Petrella, Francesco Todaro, Sabino De Gisi, Claudia Vitone, Rossella Petti, Michele Notarnicola

**Affiliations:** 1 Department of Civil, Environmental, Land, Building Engineering and Chemistry (DICATECh), Polytechnic University of Bari, Via E. Orabona 4, 70125 Bari, Italy; p.yadav@phd.poliba.it (P.Y.); claudia.vitone@poliba.it (C.V.); rossella.petti@poliba.it (R.P.); michele.notarnicola@poliba.it (M.N.); 2 Department of Industrial Engineering, University of Salerno, Via Giovanni Paolo II, 132, 84084 Fisciano, Italy; sdegisi@unisa.it

**Keywords:** contaminated sediments, industrial wastes, potential toxic elements, sustainable remediation, resource utilization

## Abstract

The routine dredging of waterways produces huge volumes of sediments. Handling contaminated dredged sediments poses significant and diverse challenges around the world. In recent years, novel and sustainable ex situ remediation technologies for contaminated sediments have been developed and applied. This review article focuses on cement-based binders in stabilizing contaminants through the stabilization/solidification (S/S) technique and the utilization of contaminated sediments as a resource. Through S/S techniques, heavy metals can be solidified and stabilized in dense and durable solid matrices, reducing their permeability and restricting their release into the environment. Industrial by-products like red mud (RM), soda residue (SR), pulverized fly ash (PFA), and alkaline granulated blast furnace slag (GGBS) can immobilize heavy metal ions such as lead, zinc, cadmium, copper, and chromium by precipitation. However, in a strong alkali environment, certain heavy metal ions might dissolve again. To address this, immobilization in low pH media can be achieved using materials like GGBS, metakaolin (MK), and incinerated sewage sludge ash (ISSA). Additionally, heavy metals can be also immobilized through the formation of silicate gels and ettringites during pozzolanic reactions by mechanisms such as adsorption, ion exchanges, and encapsulation. It is foreseeable that, in the future, the scientific community will increasingly turn towards multidisciplinary studies on novel materials, also after an evaluation of the effects on long-term heavy metal stabilization.

## 1. Introduction

Continuous dredging of harbors is necessary to maintain sufficient depth for navigational access. Dredging generates more than 600 million m^3^ of marine sediments worldwide, and in Europe, about 200 million m^3^ of sediments annually [1,2] are considered as waste materials, of which less than 1% is currently recycled. Over the past few decades, due to urban development, heavily polluted industrial enterprises, mining, and rapid economic growth, sediments have been generally contaminated by various amounts of inorganic constituents such as heavy metals, including sulfate, chlorides, and nitrate, and organic components, including polycyclic aromatic hydrocarbons (PAHs) and polychlorinated biphenyls (PCBs) [3,4]. The high concentration of toxins poses a risk of harming natural ecosystem services but also damages human health through the food chain.

Over the years, various remediation techniques have been employed within the realm of sustainable development for remediating soils/sediments contaminated with heavy metals (HMs). Among these, stabilization/solidification (S/S) technology stands out as the most well-developed method for both in situ and ex situ soil remediation applications [4,5]. According to the U.S. Environmental Protection Agency (EPA), solidification is defined as “a process that encapsulates waste into a solid material”. The mechanism involved in S/S requires the contaminants to be converted to their soluble/toxic form by reducing the surface area of the solidified materials, enveloping them with low-permeability materials, and integrating them through chemical interaction and mechanical bonding, thereby reducing the leaching concentration of the heavy metal ions [4,6]. Consequently, S/S is a key technology for the production of new materials used in civil engineering construction.

Traditionally, Portland cement and lime-based binding materials are often used due to their properties and low cost. However, cement production is associated with the emission of abundant carbon dioxide (CO_2_) and other greenhouse gasses. The cement industry plays a significant role in emissions, accounting for an estimated value of 1.45 Gigatons, or approximately 7–8% of total global CO_2_ emissions. The industry is encountering challenges in reducing CO_2_ emissions, as approximately 50–60% of CO_2_ emissions in cement production are attributed to the high-temperature calcination of limestone at 1400–1450 °C [7,8]. 

Therefore, finding new low-carbon alternatives is an important way to reduce CO_2_ release. Supplementary cementitious materials (SCMs) are being evaluated as an alternative to OPC. Ground granulated blast furnace slag (GGBS), pulverized fly ash (PFA), silica fume (SF), metakaolin, soda residue (SR), incinerated sewage sludge ash (ISSA), and various other alkaline activators like Ca(OH)_2,_ NaOH, and KOH are being used to partially substitute OPC [9,10]. OPC-based binders, when compared to bare OPC, can help in recycling waste and decreasing CO_2_ emissions in the environmental ecosystem. The incorporation of SCMs may facilitate the formation of additional calcium silicate hydrate (C-S-H) gels through pozzolanic reactions and contribute to enhance the mechanical strength, long-term durability, and immobilization of contaminants [9,11,12,13]. Researchers have employed the S/S technique with cement, lime, and other binders to mitigate the environmental impact of various wastes, including sediments. This approach aims to reduce toxicity and enhance strength properties before disposal, leveraging the abundance and benefits of renewable resources, and the presence of chlorides and sulfates, and clay compromises the strength and durability of cement products. However, recent studies have shown promising results in addressing this issue by altering the initial fluid flow state of sediments, removing hazardous substances and enhancing mechanical performance. These findings have led to the production of new materials such as filling materials, partition blocks, and paving blocks, offering a solution to the shortage of high-quality resources [4,11].

Furthermore, researchers are currently exploring the potential of using waste materials for parts of OPC in order to reduce CO_2_ emissions, reduce costs, and promote environmentally friendly practices, while maintaining long-term stability and compatibility. When sediment blocks were prepared with cement, fly ash, and lime, their compressive strength after 28 days only allowed for them to be used as filling material [12]. However, the findings from a subsequent study show that blocks made with pre-treated sediments through oxidation and thermal processes, and then further cured with CO_2_ curing and then oven dried, exhibited an improved strength of approximately 6–7 MPa, indicating their potential for use as load-bearing and non-load-bearing masonry blocks [9]. Fly ash, when combined with cement or calcium oxide, behaves as both a filler and an artificial pozzolana, enhancing the effectiveness of the treatment [13]. A study was conducted using a blend of cement and seashell powder to treat lead-contaminated soils, incorporating wood-based biochar [14]. 

This study aims to assess the immobilization of heavy metals in marine sediments using various OPC-based binders, summarizing the materials, conditions, and effects based on existing research. The effects of heavy metals can be determined through the leaching concentration and the compressive strength of the products. On this basis, this review prospects the relevant studies of OPC-based binders and further proposes future research directions.

## 2. Principles of S/S Technology

The contaminants in dredged marine sediments depend on the location chosen for investigation or sampling. Generally, these sediments contain natural organic and mineral elements resulting from coastal erosion, including both organic and inorganic pollutants [4,15] and heavy metals such as lead (Pb), chromium (Cr), mercury (Hg), zinc (Zn), cadmium (Cd), copper (Cu), and salts [12]. The effective remediation of heavy metals involves stabilization/solidification (S/S) technology, which has been developed for both in situ and ex situ soil remediation. This technology primarily encompasses three approaches: (1) surface adsorption, (2) chemical bonding, and (3) physical encapsulation [5,16]. During the process of adsorption, the surface and pore structures of C-S-H, C-A-H, and C-A-S-H gels attract heavy metal ions without undergoing any chemical reaction. Chemical bonding involves reactions between heavy metal ions and hydrate gels, such as precipitation in an alkaline environment and ion exchanges. The physical encapsulation process involves the precipitation or filling of the products’ pores. Heavy metals can be classified as amphoteric-type, precipitation-type, and anion-type [4].

Traditionally, Ordinary Portland Cement (OPC) has been extensively used as a mature and reliable material for stabilization/solidification applications due to its alkaline properties and strength. Nonetheless, cement production leads to increased greenhouse gas emissions and energy requirements. Carbon dioxide emissions from the cement industry to produce OPC is approximately 12% of all industrial CO_2_ emissions [7,9], making it important to focus on developing more eco-friendly or low-carbon alternatives.

### 2.1. Waste Resources Combined with OPC

Based on the calcium/silica ratio, SCMs can be classified into two groups: hydraulic materials (containing calcium and reacting with H_2_O) and pozzolanic materials (having low or zero cementitious properties but reacting with Ca(OH)_2_). Most SCMs possess hydraulic or pozzolanic properties [17,18].

#### 2.1.1. Pulverized Fly Ash

Pulverized fly ash (PFA) is a residual product obtained from the flue gas of bituminous coal powder furnaces by electrostatic and mechanical processes [16]. The primary components of PFA are SiO_2_, CaO, Al_2_O_3_, MgO, and some carbon as a residue, which closely resembles the composition of OPC [13]. As a result, PFA has been used as an effective stabilizer in recent decades. It offers lower energy consumption and presents a more economical way for heavy metal removal in the S/S treatment process [16,19]. 

PFA is classified into class-C and class-F PFA based on high and low calcium (Ca) contents [16]. Therefore, the use of both types depends on the need for alkali. Class-C PFA can provide additional alkali for the precipitation of metals, while class-F PFA produces a secondary C-S-H gel for the immobilization of metals through a pozzolanic reaction [13]. A semi-dynamic leaching test was carried out to investigate the influence of cement-based binders with waste materials on the long-term leaching characteristics of geogenic As. The findings indicate that the presence of cement and PFA led to a significant increase in leached arsenic (As) ions. Furthermore, mineral additives with higher Ca content and pozzolanic activity were more effective in reducing the leached As concentrations. Class-F PFA is an appropriate material when combined with OPC for binding. Contrary to arsenic (As), cadmium and lead have lower leaching concentrations, respectively, at pH 9 and 11 [20]. Additionally, for Pb-contaminated soil, the S/S technique was utilized for class-C fly ash and soda residue, and it was concluded that fly ash demonstrated a better immobilization potential in S/S than soda residue [13]. The spherical-shaped particles of PFA can improve flowability and strength through pozzolanic reactions, as well as micro-aggregate filling and rolling friction. Hence, PFA is a more widely used alternative to ordinary Portland cement in the S/S of contaminated sediments [9].

#### 2.1.2. Soda Residue

Soda residue (SR) is an alkaline solid waste produced during the manufacturing of sodium carbonate as a by-product of the ammonia–soda process, which is an alkaline solid waste with a pH ranging from 11 to 12. SR primarily consists of calcium salts like CaCl_2_, Ca(OH)_2_, and CaCO_3_, together with calcium, silicon, magnesium, aluminum, and some silicon and iron oxides [21]. The most important characteristics of SR are durability and porosity, attributed to the presence of a fine particle size that is smaller than 0.074 mm, leading to a high specific surface area that enables the adsorption of more heavy metal ions in the S/S system [13]. The composition of SR is similar to OPC, with both containing Ca, Si, and metal compounds, and both are viable sustainable alternatives to partially replace OPC. Testing was conducted on the mechanical properties of soda residue soil in both laboratory and field settings using various combinations of SR and fly ash (FA) to explore the reutilization of waste SR and its potential application. The findings demonstrate that the chemical composition of SR consisted of insoluble salts, and the cohesive force, internal friction angle, and UCS of SR are 40 kPa, 15.6°, and 0.02 kPa, respectively. Additionally, the inclusion of FA contributed to the enhancement of soda residue strength, with the incorporation of approximately 50% FA resulting in the highest cohesive force, internal friction angle, and UCS, namely, 74 kPa, 32°, and 0.43 kPa, respectively [22]. 

For heavy metal stability, cement–soda residue has proven to be an effective binder for S/S due to its alkaline nature. An investigation on the leaching behavior of Zn-contaminated samples treated with cement–soda residue under acid rain conditions, using flexible wall leaching test [23], determined the concentrations of Zn^2+^ and Ca^2+^ in the filtrate. The Zn^2+^ concentration in the filtrate was compiled with the third-grade applicable standard of <1 mg/L, according to the Chinese National Environmental Quality standard. Samples treated with SR exhibited higher strength compared to PFA with a high calcium content, and freeze-thaw resistance was further enhanced by adjusting the proportions of OPC and SR [22,24].

#### 2.1.3. Ground Granulated Blast Furnace Slag (GGBS)

Ground Granulated Blast Furnace Slag (GGBS) is derived from the iron and steel industry and is widely acknowledged as a suitable partial substitute of cement. It can be used in premixed concrete, bulk on-site concrete production, and the creation of precast elements [16,25]. GGBS generates low heat of hydration during the hydration process, interacts with Ca(OH)_2_, and forms an additional C-S-H gel, which improves the heavy metal immobilization and mechanical strength of the S/S products [25]. However, GGBS also acts as a filler to densify the pore structures and facilitate low permeability. The main chemical compositions of GGBS are CaO = 41.1 wt% of the total components and SiO_2_ = 34.11 wt%. Additionally, Al_2_O_3_ and MgO accounted for 11.16 wt% and 6.57 wt%, respectively, with other contents contributing less than 1% by weight. As GGBS is rich in Al_2_O_3_ (10–20%), sediments treated with GGBS exhibited higher strength, as shown by a comparison of the unconfined compressive strength (UCS) of OPC-treated samples (350 kPa) and GGBS-mixed OPC-treated samples (945 kPa), revealing that the addition of GGBS made UCS almost 2.7 times higher at the curing time of 56 days [26]. Further studies also demonstrated that incorporating GGBS with OPC is effective in the immobilization of heavy metals and also offers resilience against sulfate and chloride compounds in contaminated sediments [16,27]. 

#### 2.1.4. Silica Fume (SF) 

Silica fume is a silica-rich material and contains as much as 99% reactive SiO_2_. Ordinary Portland cement undergoes hydration, thus generating hydration products such as the C-S-H gel and Ca(OH)_2_. The addition of silica fumes to cement results in its reaction with Ca(OH)_2_ and leads to the formation of an additional C-S-H gel through a pozzolanic reaction. This newly formed C-S-H gel exhibits greater resistance to aggressive chemicals than calcium hydroxide and increases the durability of the solidified/stabilized products. However, some heavy metals were dissolved in a high pH OPC environment. The incorporation of SF reduces the pH of the solutions and demonstrates favorable immobilization properties, as most metals have a lower solubility in the pH range of around 11–13 [28]. The particle sizes of SF are 100–150 times smaller compared to OPC, which leads to better particle packing, fills the pores created by free water, and refines the microstructure of the product by forming a dense pore structure and reducing the leachability of toxic elements [16,29]. To investigate the potential use and effectiveness of expansive clay stabilization using cement and silica fumes, a blend with a 10% cement replacement was found to reduce the curing period for the successful treatment of swelling clay. Additionally, it resulted in a 35% increase in strength and a 50% decrease in the compression index compared to using sole cement [29]. 

Thus, the incorporation of SF into the binding system can significantly reduce the leaching of toxic elements and can also enhance the strength of products for engineering applications [28,29].

#### 2.1.5. Metakaolin (MK) 

Metakaolin is a product of kaolin clay that is formed at a temperature of 500–800 °C, and 99.9% of its particles are <16 μm, with a mean particle size of about 3 μm [30]. In comparison to fly ash, MK offers a more consistent chemical composition and is primarily composed of the oxide components Al_2_O_3_ and SiO_2_. Its presence accelerates the hydration reaction in the cement, fills pores, enhances the final strength of the products, reduces permeability and shrinkage through particle packing, and contributes to the formation of denser concrete. Studies have indicated that metals such as Pb^2+^, Cu^2+^, Cd^2+^, and Cr^3+^ can be effectively immobilized in MK-based geopolymers [31]. Tests were conducted on metakaolin to investigate its impact and the effects of different ions on the mechanical properties of salt-rich soil–cement, revealing that the strength of the soil–cement increased as the metakaolin content increased [32]. Subsequently, in a study on metakaolin and fly-ash based polymer concrete, where fly ash and metakaolin were mixed in a 1:1 mass ratio and potassium silicate was used as an activator, a 93.7% increase in compressive strength after 3 days of curing and a 134.4% increase after 7 days were demonstrated [31]. It was also concluded that by increasing the ratio of metakaolin to fly ash, a significative enhancement of the structural density and compactness of the mortar/concrete was observed.

#### 2.1.6. Incinerated Sewage Sludge Ash (ISSA)

Incinerated sewage sludge ash (ISSA) is a by-product of sludge incineration plants. It shows pozzolanic properties that are attributed to its alumina and silica content and is composed of SiO_2_, Fe_2_O_3_, Al_2_O_3_, CaO, Na_2_O, and MgO [27,33]. ISSA can serve as an OPC substitute in cement-based S/S to remove heavy metals during the hydration process by incorporating an interlocking structure due to its extensive surface area and porosity [16]. The inclusion of ISSA can enhance treatment characteristics and reduce environmental impacts through both chemical and physical adsorption. The treatment of lead (Pb)-contaminated soils with ordinary Portland cement (OPC) and blended OPC containing incinerated sewage sludge ash (ISSA) resulted in the leachability of Pb that was regulated by the combined influence of adsorption, encapsulation, or precipitation in S/S soils [27]. Contrary to this, samples treated with ISSA exhibited a lower unconfined compressive strength compared to GGBS-treated samples [27,33]. However, the incorporation of ISSA and GGBS together resulted in favorable mechanical strengths [10]. 

#### 2.1.7. Alkaline Activators Cement

Alkali-activated cements (AACs) are promising binders for civil and environmental engineering applications and can be used as a substitute either in part or as a whole, depending on the availability of local raw materials. The strength of soil products treated with OPC gradually increases. However, the addition of certain alkaline activators like alkali hydroxide, sulfate, and sodium silicate can accelerate the hydration process and enhance strength development [34]. Based on previous research, it has been observed that alkali hydroxides such as Ca(OH)_2_, NaOH, and KOH can react with OPC without requiring additional additives to prevent soil product expansions. Research indicates that alkali-activated cement can immobilize toxic elements such as Zn^2+^, Pb^2+^, Cd^2+^, and Cr^6+^, which could be immobilized in NaOH, Na_2_CO_3_, and sodium–silicate-activated slag cement [35,36] through a series of reactions like adsorption, physical immobilization, ion exchange, neutralization, precipitation, reduction, complex formation, and a lower S^2−^ content in a blast furnace slag support to stabilize Cr^6+^. Compared to OPC-based binders, AACs exhibited superior properties, including reduced permeability and improved resistance to acid and sulfate [37]. The combination of fly ash (FA) and sodium hydroxide (NaOH) was used to enhance the mechanical strength of the compressed earth blocks (CEBs) manufactured from dredged sediments by partially replacing the sediment with fly ash from 10% to 50%. This resulted in a significant improvement in the dry and wet mechanical strength of CEBs, with the maximum strengths reaching 9.0 MPa and 6.9 MPa in dry and wet conditions, respectively [38]. Additionally, a metakaolin-based geopolymer (MKG) was employed to stabilize synthetic lean, revealing that MKG could improve the mechanical properties of soil products containing high sulfate levels.

#### 2.1.8. Seashells

Seashells, deriving from the fishery industry as bio-waste, are non-biodegradable materials due to their calcium carbonate (CaCO_3_) composition, with a mineral phase of calcite [39]. The primary chemical composition of seashells is similar to limestone, primarily consisting of calcium oxide (CaO) with small fractions of other oxides. In the case of oyster shells, the CaO composition ranges from 48.0% to 86.8%, with a high loss on ignition (LOI) varying between 23.2% and 51.0% [40]. Table 1 provides a summary of the CaO content and LOI for seashells used in various research studies. The variations in the CaO content reported by different researchers may be attributed to the differences in the temperature used for the calcination. For instance, some research studies found a high CaO content of 87.2% in mussel shells after calcining at a temperature of 1100 °C, whereas others observed CaO content of about 53.0% [41,42]. Seashells have been investigated for a variety of applications, including the removal of heavy metals and as substitutes for traditional materials like cement, sand, and coarse aggregates [39,43,44]. In this context, a recent project funded by the LIFE programme (Project: 101114177–LIFE22-ENV-IT-LIFE GREENLIFE4SEAS, i.e., GREen ENgineering solutions: a new LIFE for SEdiments And Shells, https://greenlife4seas.poliba.it/ (accessed on 19 June 2024)) will produce in situ breakwaters, outdoor paving blocks, and mass stabilization by treating dredged sediment with a reduced amount of cement, as it is partially replaced by a powder produced from non-calcined mussel shells. 

Additionally, mussel shell ash was found to have a pH of >12 and a high electrical conductivity of 16.01 to 27.27 dS/m, while the calcined shell exhibited pH values of up to 10.7 and electrical conductivities between 1.19 and 3.55 dS/m [45]. The combination of mussel shell calcination ash, sewage sludge, and wood ash resulted in an excellent immobilization of Hg and As, with adsorption rates of around 99% for Hg and 90–96% for arsenic (As) and a 32% adsorption for Cr [46]. When used to stabilize lead (Pb)- and copper (Cu)-contaminated soil from a firing range, the synergistic effect of 10 wt% calcinated oyster shells (COSs) and 5 wt% FA resulted in a significant reduction in leachability of Pb (>98%) and Cu (>96%), whereas the addition of only FA did not effectively reduce the leachability of Pb and Cu [47]. The immobilization of metal ions was strongly associated with ettringite and pozzolanic reaction products.

**Table 1:** Calcium oxide (CaO) composition and LOI of seashells.

Seashell Types	Authors	CaO (wt%)	LOI (wt%)
Oyster shells	Lertwattanaruk et al. [42]	53.6	42.8
	Kuo et al. [48]	77.8	-
	Li et al. [49]	86.8	-
	Djobo et al. [40]	74.7	23.2
Mussel shells	Petti et al. [44]	53.61	45.58
	Leone et al. [39]	52.21	44.91
	Lertwattanaruk et al. [42]	53.4	42.2
	Felipe-Sese et al. [50]	87.2	-
	Yao et al. [41]	53.7	45.6
Clam shells	Lertwattanaruk et al. [42]	54.0	42.7
	Olivia et al. [51]	67.7	-
Cockle shells	Lertwattanaruk et al. [42]	54.2	42.7
	Olivia et al. [51]	51.9	-
Seashells not specified	Soltanzadeh et al. [19]	52.34	41.25

### 2.2. Cement Hydration

**Figure 1:**
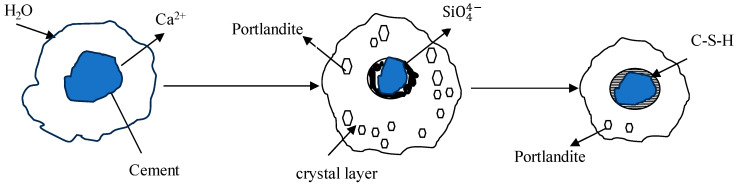
Gel model of cement hydration [52,54,55].

**Figure 2:**
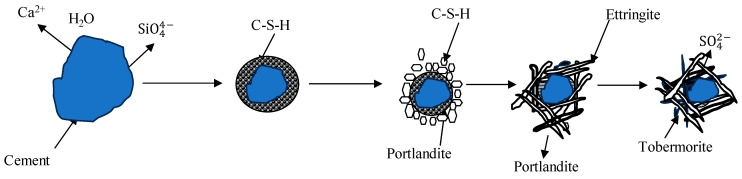
Crystal model of cement hydration [52,54,55].

As OPC-based binders and soil/sediments are composed of silicon oxide and calcium compounds, these dissolve and generate free silicon dioxide and calcium ions and form a C-S-H gel membrane during hydration. The formation of a membrane allows for the inward flow of water and outward migration of Ca^2+^ and silicate ions because of different osmotic potentials on both the inward and outward sides of the membrane, as shown in Figure 1 and Figure 2. Portlandite Ca(OH)_2_ will accumulate on the waterside [52]. The pozzolanic reactions between Ca^2+^, SiO_2_, and Al_2_O_3_ occur in an alkaline environment and form C-S-H, C-A-H, and C-A-S-H. The hydration reactions producing calcium hydroxide were studied by [53,54,55] and are reported below.

3CaO⋅SiO_2_ + nH_2_O → xCaO⋅SiO_2_⋅(n − 3 + x)H_2_O + (3 − x)Ca(OH)_2_ (1)

2CaO⋅SiO_2_ + nH_2_O → xCaO⋅SiO_2_⋅(n − 2 + x)H_2_O + (2 − x)Ca(OH)_2_ (2)

The strength of solidified contaminated soils can be ascribed to the micro-aggregate filling effect, the morphological water-reduction effect, and the pozzolanic activity effect. The hydration process increases pH in the cement/soil interaction, and ionization of calcium hydroxide occurs (pH rises to >12.4).

Ca(OH)_2_ → Ca^2+^ + 2(OH)^−^ (3)

The high pH of the reaction influences the solubility of clay minerals, as reported in the following Equations (4) and (5).

(4) $${\mathrm{Al}}_{2}{\mathrm{Si}}_{4}O_{10}{\left(\mathrm{OH}\right)}_{2}\cdot {\mathrm{nH}}_{2}O+2{\left(\mathrm{OH}\right)}^{-}+10H_{2}O\;\to \;2\{2\mathrm{Al}{\left(OH\right)}_{4}^{-}+4H_{4}{\mathrm{SiO}}_{4}\}+{\mathrm{nH}}_{2}O$$

(5) $$2H_{4}SiO_{4}\to 2H_{3}SiO_{4}^{-}+2H^{+}\to 2H_{2}SiO_{4}^{2-}+2H^{+}$$

Then, the ions of Si and Al compounds interact with the Ca ions released from Ca(OH)_2_, as follows from Equations (6) and (7).

(6) $$H_{2}SiO_{4}^{2-}+Ca^{2+}+2OH^{-}\to C-S-H\{3CaO\cdot 2SiO_{2}\cdot 3H_{2}O\}$$

(7) $$Al{\left(OH\right)}_{4}^{-}+Ca^{2+}+2OH^{-}\to C-A-H\{3CaO\cdot Al_{2}O_{3}\cdot Ca{\left(OH\right)}_{2}\cdot 12H_{2}O\}$$

The formation of a C-A-S-H gel is produced by silica and alumina ions and calcium hydroxide, as follows (8).

(8) $$2SiO_{4}^{2-}+Al{\left(OH\right)}_{4}^{-}+Ca^{2+}+2OH^{-}\to C-A-S-H\{CaO\cdot Al_{2}O_{3}\cdot 2SiO_{2}\cdot 4H_{2}O\}$$

The presence of $SO_{4}^{2-}$ in OPC-based binders and sediments allows for a reaction with C-A-H, resulting in the formation of ettringite (Aft) crystals during the early stages of hydration. AFt can effectively capture heavy metals through chemical adsorption and can react with CO_2_ to produce calcite, which helps to fill pores and improve structural stability [5,56]. As hydration progresses, the sulfate concentration decreases, causing more AFts to aggregate and transform into calcium monosulfoaluminate hydrates (AFms). This transformation can weaken the product due to a lower insoluble CaSO_4_ content, as shown below from reactions 9 and 10 [54].

(9) $$C_{3}A+3CaSO_{4}\cdot 2H_{2}O+26H_{2}O\to 3CaO\cdot Al_{2}O_{3}\cdot 3CaSO_{4}\cdot 32H_{2}O$$

(10) $$Al_{2}O_{3}+3Ca{\left(OH\right)}_{2}+3\left(CaSO_{4}\cdot 2H_{2}O\right)+23H_{2}O\to 3CaO\cdot Al_{2}O_{3}\cdot 3CaSo_{4}\cdot 32H_{2}O$$

Adding limestone can reduce the conversion of Aft to AFm, leading to the formation of stable mono-carbonate in place of mono-sulfate [24]. Ettringite is one of the main components of expansion, resistance against shrinkage, rapid hardening, and early strength development. The modernization of cements may have caused an increase in the presence of ettringite due to the additional sulfate, which assists in controlling the set time of clinkers and enhancing early strengths [57]. The reaction of desulfurization gypsum with C_3_A in cement leads to the formation of ettringite (AFt) crystals. Additionally, under alkali conditions, desulfurization gypsum reacts with Al_2_O_3_ as a sulfate activator to form AFt. AFt has the ability to trap metals such as Cu, Cr, Cd, Pb, Zn, and Fe through a cationic substitution within its lattice structure [5,54].

## 3. Mechanism of S/S for Heavy Metals 

Sediments usually contain heavy metals such as arsenic (As), cadmium (Cd), chromium (Cr), copper (Cu), mercury (Hg), nickel (Ni), lead (Pb), zinc (Zn), etc. For the remediation of heavy metals, the promising tool of stabilization/solidification (S/S) technology [4,5] mainly includes (1) physical adsorption, (2) chemical fixation, and (3) physical encapsulation [58]. In the case of adsorption, the heavy metal ions adhere to the surface of the hydration products (i.e., on the pore structure of C-S-H, C-A-H, and C-A-S-H gels without any chemical reaction) [5]. Chemical fixation means that reactions take place between the heavy metal ions and the hydrate gels, such as precipitation in an alkaline environment and ion exchanges [59]. The physical encapsulation process refers to the precipitation or filling of the pores’ products. Heavy metals can be divided into amphoteric, precipitation and anion types [4,53]. 

The calcium silicate hydrate gel (C-S-H) is a mixture of crystallized particles with different morphologies that are classified into four types: (1) fibrous, (2) a reticular network, (3) accumulated grain morphology, and (4) inner product morphology. Type 1 and 2 are early-stage hydration products and form a honeycomb or reticular network, whereas types 3 and 4 are fairly massive and appear only in older pastes of mortar/concrete [57,60,61]. The C-S-H gel contains the bulk of micro-pores, provides a high surface area, and largely controls the sorption properties. It was also reported that the C–S–H gel had an excellent capacity to bind metals. The carbonation of C_3_S resulted in an enhanced capacity of adsorbing heavy metal cations and hydroxyl ions because of the large surface area of the C–S–H gel and calcium carbonate [5,62]. The double electrostatic layer, triple electrostatic layer, and charge dispersal models were developed to explain the intrinsic mechanisms involved in the S/S process. The triple electrostatic layer model suggests that Ca^2+^ ions from initial hydration create a tightly bound bi-layer with the negatively charged C–S–H gel surface. Afterwards, heavy metal cations and hydroxyl anions quickly align with the bi-layer to form a tri-layer [52]. The charge dispersal model illustrates that Ca^2+^ ions encircle the negatively charged C–S–H surface and selectively adsorb to generate a positive-charge layer, while other complex ions disperse around the surface. The adsorption of heavy metals hinders the uniform nucleation or growth of hydration products in some cases, and in other cases, it promotes silicate polymerization [63].

The performance of cement-based S/S systems [4,53,57,58] were investigated by mixing heavy metal hydroxides of Pb, Zn, and Cu with tricalcium silicate (C_3_S) and tricalcium aluminate (C_3_A), which were then blended with OPC [58]. All heavy metal hydroxides, Zn(OH)_2_, Pb(OH)_2_, and Cu(OH)_2_, adversely affected the hydration of C_3_A, but Zn(OH)_2_ can completely inhibit C_3_S hydration due to the formation of CaZn_2_(OH)_6_·2H_2_O [5,52]. The reactions are as follows.

(11) $$Zn^{2+}+2OH^{-}\to Zn{\left(OH\right)}_{2}$$

(12) $$Zn{\left(OH\right)}_{2}+OH^{-}\to H_{2}O+{ZnO}_{2}^{2-}$$

(13) $$2ZnO_{2}^{2-}+C_{3}S/O-{Ca}^{2+}+6H_{2}O\to C_{3}S/O-{CaZn}_{2}{\left(OH\right)}_{6}\cdot 2H_{2}O+2{OH}^{-}$$

Cu_6_Al_2_O_8_CO_3_·12H_2_O, Pb_2_Al_4_O_4_(CO_3_)_4_·7H_2_O, and Zn_6_Al_2_O_8_CO_3_·12H_2_O were formed in the samples containing C_3_A, and the addition of CaSO_4_ in C_3_A increases the detrimental effects of heavy metals due to the calcium aluminate sulfates and heavy metal aluminate carbonates. The interaction between different heavy metal ions and various binders differs in immobilizing Cr^3+^ in soil or sediments, where they can substitute Si^4+^ and Ca^2+^ in the C-S-H gel; replace Al^3+^ in the C-A-H gel; and displace Al^3+^ in ettringite during hydration reactions, ion exchanges, and pozzolanic reactions, resulting in the creation of intricate precipitates such as Ca_2_Cr(OH)_7_⋅3H_2_O and Ca_2_Cr_2_O_5_⋅6H_2_O [23,58,59]. As a result, the strength of the stabilized samples initially contaminated with Cr^3+^ was significantly compromised compared to the soils contaminated with Pb^2+^ and Zn^2+^ [23,53]. Furthermore, heavy metals can be also absorbed by hydrated products, iron oxides, and hydroxides present in the cement [27]. 

The combination of red mud and cement in a 3-to-1 ratio effectively immobilized Pb compared to Zn and Cu, due to the formation of absorption sites on the surface of iron (Fe) and aluminum (Al) oxides [58]. In addition, the use of lime, organo-clay, and activated carbon to recycle contaminated marine sediments (CMSs) failed a leaching test for copper at 28 days, but with an extended air curing period of 56 days, all the investigated metals were stabilized [4]. In addition, the presence of Ni and Cu in the soil/sediments would determine a reaction with Ca(OH)_2_, forming Cu(OH)_2_ and CaNi(OH)_6_·2H_2_O [58]. Although, OPC-based binders can utilize waste, and the effect on the leaching concentration and strength of the products differ with various OPC-based binders. Table 2 summarizes recent studies on the leaching concentration and strength effects of HMs in S/S.

## 4. Influence of Chloride and Sulfate Ions

Due to the hazardous matter in the marine dredged sediments (MDS) and marine environment, the chemical attack on the marine sediment concrete/mortar is a major concern.

*(I)*  *Chloride Attack*

The presence of chloride ions is one of the threats affecting the durability of concrete. Generally, the steel reinforcement will form a passive layer on its surface due to the high pH of concrete. Continuing de-passivation under a complex chloride concentration and carbonation process can cause free chloride ions in marine sediment concrete (MSC) to initiate an exfoliation of the concrete cover and a corrosion of the steel reinforcement through the following chemical reactions [65,66].

(14) $${Fe}^{2+}+2{Cl}^{-}\to {FeCl}_{2}$$

(15) $${FeCl}_{2}+2H_{2}O\to Fe{\left(OH\right)}_{2}+2HCl$$

Chloride permeability and chloride migration can provide information on the chloride attack. However, the chloride penetration rate does not only define the resistance. Chloride permeability depends on the degree of hydration, curing conditions, the mix design, and the use of admixtures. The key factors affecting permeability and chloride ion diffusion are associated with the distribution of the pores in the portion of the cement paste, as the size and connectivity of the composite must be taken into account when determining the aggressive medium penetration in the concrete [67]. Moreover, the chloride ions can react with C_3_A and C_4_AF to form Friedel’s salt, which can expand to fill the pores, is unstable, and tends to be soluble with a decrease in pH [67,68]. This mechanism will not be strong enough to improve the compactness, at an excessively high porosity, and chloride ions can penetrate it. Chloride ions in MDS can reduce carbonation by forming Friedel’s salt, and because of the increased carbon dioxide, the dissolution of Friedel’s salt will start, as CO_2_ can react with $OH^{-}$ and Al(OH)_3_ and leads to dissolution [68,69], as shown below:

(16) $$CaO\cdot Al_{2}O_{3}\cdot 3{CaCl}_{2}\cdot 10H_{2}O\to 6Ca^{2+}+6Cl^{-}+6OH^{-}+2Al{\left(OH\right)}_{3}+4H_{2}O$$

This may lead to free chloride ions and may limit carbonation, but carbonation can release more free chloride ions and facilitate chloride erosion.

*(II)*  *Sulfate Attack*

A sulfate attack generally corrodes concrete and affects the durability of concrete subjected to aggressive environments. Sulfate intrusion leads to the loss of concrete strength and mass and penetrates through capillary pores in the form of sodium sulfate and magnesium sulfate [65]. The mechanism of deterioration of the concrete depends on several factors: the temperature, the associated cation, the sulfate ion concentration, and also the roles of tricalcium aluminate (C_3_A) and portlandite (Ca(OH)_2_) [70]. As defined in EN 197-1 [71], a Portland cement CEMI with 3% C3A, CEMIIB, and CEMIIIC is considered resistant to sulfates. 

Since ettringite and gypsum have a swelling tendency, the continuous growth of gypsum and ettringite will create a volumetric expansion and expansion stress and will lead to cracks in the concrete.

## 5. Water Content and Curing

The product’s strength increases with the curing time due to the formation of more gels and other compounds. However, the water-to-cement or water-to-binder ratio (w/c or w/b) also affects the fluidity and workability of the mortar/concrete. Simultaneously, the change in the w/c ratio also affects the strength of products, volumetric shrinkage, bulk density, curing time, and pH [12,72]. Investigating all the mentioned points, a research study involving three different sediments from Italy and France, with varying water–cement ratios and sediment replacement percentages, while keeping a constant slump, revealed that the crucial factor in solidifying soil is a control of the moisture content [72]. Also, changes in the porosity and bulk density showed an almost linear correlation but a decline in strength and a significant increase in drying shrinkage, up to 10 times that of the reference mortar, were observed with a higher substitution of sediments with sand [72]. Subsequently, with the addition of fly ash in OPC, there was an increase in the water demand of the mix, and a decrease in the strength of the soil product was observed as the fly ash-to-OPC ratio increased [73]. This could be attributed to the position occupied by the original water being converted into voids and pores. Another research study indicated that a higher w/c ratio facilitated the mineral dissolution and accelerated the release of HMs, with a decrease in the strength of soil products [74]. On the contrary, a low water content would lead to an incomplete hydration reaction process due to undissolved reactants [4,72]. 

## 6. Sediment Utilization as Resources

**Figure 3:**
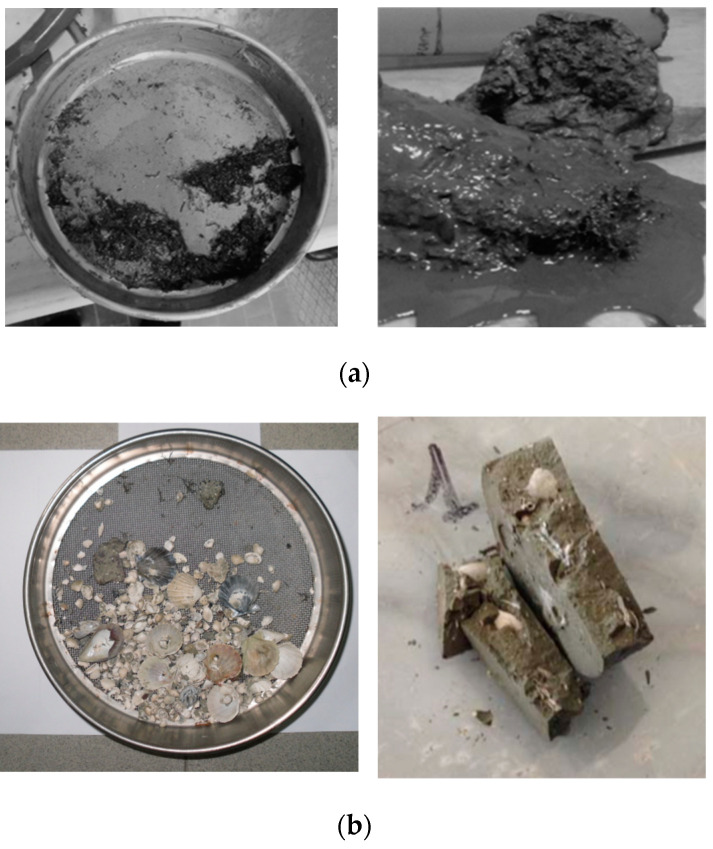

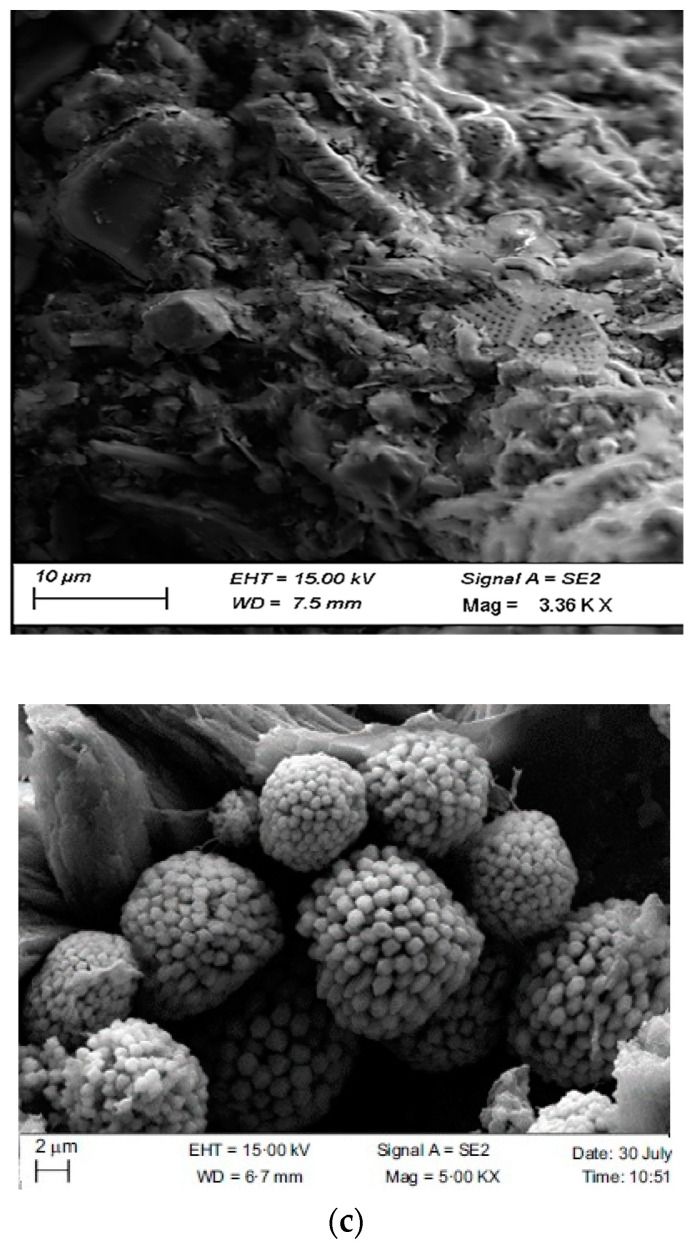
Representation of some of the complexity sources at multiple scales that are present in sediments: (**a**) organic matter found in shallow samples; (**b**) fossils and shells detected during sieving and mechanical tests; (**c**) diatoms [3] and Framboidal pyrite [44], identified through SEM images on sediments.

Sediments are natural geomaterials of high complexity (Figure 3 [3]). Such complexity stems from their heterogeneity in composition, which can vary from sandy soils and essentially clay materials to the presence of natural compounds (salinity in pore water, organic matter, diatoms, and fossils in the matrix). Moreover, when polluted, the presence of anthropogenic compounds (heavy metal ions and organic pollutants) in various concentrations can further affect their hydro-mechanical behavior, making their in situ chemo-mechanical stabilization particularly challenging. This is why there are not a lot of research contributions concerning cutting-edge options for their ex situ S/S treatments.

The ex situ treatment processes generally aim to minimize the environmental impact of the contaminated sediments by using recycling methods. The substantial presence of sand, silt, and clay in the sediment contributes to a significant value to construction materials and some economic benefits. In recent studies, contaminated sediment has been successfully transformed into fill materials, supplementary cementitious materials (SCMs), paving blocks, partition blocks, ready-mixed concrete, and other products, showing a path for the sustainable use of contaminated sediments.

### 6.1. Filling Materials

With an appropriate mix design, contaminated sediments in combination with the other wastes can be valorized into filling materials. In a recent study, a composite made with lime, organoclay, and activated carbon without pre-treatment achieved an acceptable unconfined compressive strength of 28 kPa [4], surpassing the minimum required value for slope stability (i.e., 18 kPa for a minimum factor of safety equal to 1.15 for a 3.5 m slope and 24 kPa for 5.0 m slope for fill materials of site formations [75]). The composite underwent a leaching test and failed for copper only at 28 days, but with an extended curing time of 56 days, it met the requirements for all investigated metals [4]. Afterwards, by using lime, fly ash, and sediments, it was possible to achieve a compressive strength higher than 1 MPa at 28 days, reaching the strength criteria of filling materials [12]. Subsequently, a changed curing environment from the traditional methods to CO_2_ curing conditions further increased the strength of the materials derived from sediments. When CO_2_ reacts with the cement clinker (C_3_S and C_2_S), it forms C-S-H gels, and Ca(OH)_2_ can be carbonated to form CaCO_3_ $\left(Ca{\left(OH\right)}_{2}+CO_{2}\to CaCO_{3}\right)$ The additional hydrates improved the bonding strength, and the carbonates densified the microstructure, leading to an enhanced strength of the sediment-derived products [76]. From an economical and technical point of view, recycling contaminated sediments as fill materials can be a cost-effective solution. 

The use of dredged contaminated marine sediments as aggregate fill materials can be improved by incorporating strong performance and excellent immobilization efficiency through waste-enhanced binders such as pulverized fly ash, incineration sewage sludge ash, lime, red mud organoclay, biochar, etc. [16]. Previous research has demonstrated that the addition of silica-rich waste materials enhanced the mechanical strengths and the resistances of Mg cement-based solidified filling materials by promoting the formation of a magnesium silicate hydrate (M-S-H) gel in reactive magnesium oxide (MgO) cement [77,78]. However, the incorporation of metakaolin, red mud, and blast furnace slag with cement for the remediation of arsenic (As)-contaminated sediment showed that the binder with a higher Ca content facilitated the formation of calcium and arsenic (Ca–As) complexes, while binders enriched with iron strongly aided in the formation of iron–arsenic complex compounds and demonstrated high effectiveness in immobilizing As, reaching around a 99.9% efficiency [64]. On the contrary, the incorporation of dredged marine sediments pushed for a higher water demand because of the fine particle size, requiring measures to maintain the appropriate workability and rheological properties of sediment-derived products [72,79]. 

### 6.2. Partition/Paving Blocks

**Figure 4:**
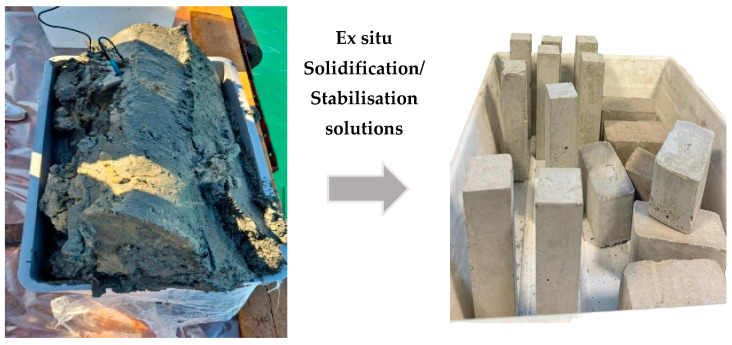
From dredged sediments to prototypes of pavement blocks.

Sediments with or without any pre-treatment can be used for the preparation of blocks (Figure 4) through different methods by considering the level of contaminants, water content, and particle size distribution [11]. Pavement blocks were prepared on an industrial scale using non-polluted marine sediments at a substitution ratio of 19% to partially replace quartz sand. The reported splitting tensile strength of 3.58 MPa was very close to the standards and had a lower water absorption ratio (4.05%) than ordinary paving blocks. Additionally, leaching findings demonstrate that the quantities of HMs that were extracted from crushed paving blocks were within the regulatory limits [80]. A study on the long-lasting properties of a cement mixture made up of treated sediments with varying levels of cement substitutions (0%, 10%, 20%, and 30%) revealed that the mortar containing a 10%-treated sediment performed as effectively and durably as the standard mortar [65]. With the use of marine sediments as a binding agent, high-strength blocks achieved a mechanical strength of 14.5 MPa at 90 days, with a higher substitution rate of 50–60 weight percentage [81]. Dredged marine sediments as aggregates were used to produce pavement and partition blocks. Meanwhile, pollutants in the sediments were efficiently immobilized to meet the standard acceptance limits of leaching [82]. Additionally, the incorporation of biochar and CO_2_ curing also enhanced the mechanical strength of sediment-derived blocks. After one day of CO_2_ curing and seven days of air curing, the compressive strength of the sediment eco brick blocks was 45 MPa [10,83,84], fulfilling the strength requirement for vehicle paving blocks. After being calcined at 850 °C, dredged marine sediments were recycled and used as a binder to fabricate clay bricks with a favorable thermal insulation capacity [65]. 

### 6.3. Ready-Mixed/Foamed Concrete

The feasibility of using dredged marine sediments for concrete production is a major concern among researchers to promote sustainable developments. By using oven-dried and sieved marine dredged sediments as fine aggregates (size < 2.36 mm) and re-saturating them to a moisture content of 60%, three groups of concrete (A, B, and C) were produced by incorporating different waste materials and cement. The results demonstrate the optimal utilization of various waste materials in concrete production for both load-bearing and non-load-bearing purposes. When the sediment replacement ratios are 40% or lower, they can be recycled for non-load-bearing construction applications, while higher replacement ratios can be employed in formation/filling works at construction sites [9,83]. Before use in ready-mixed concrete, marine sediments containing Cl^−^ and $SO_{4}^{2-}$ require pre-treatment. Additionally, ground and dried marine sediments have the potential to replace cement in concrete/mortar production. Previous research has demonstrated that a mortar with a 20% substitution of cement with ground sediment exhibits better mechanical properties compared to other mortars prepared with cement containing a similar dosage of limestone fillers [85]. Furthermore, it has been noted that sediments, as a filler, have a positive impact. However, due to the chloride content in sediments, this concrete cannot be used for reinforced concrete applications. 

Foamed concrete, a porous material made of cementitious materials, admixtures, and foaming agents, finds wide applications in construction due to its lightweight and thermal insulation properties [18]. The foamed concrete derived from sediment achieved a compressive strength of 5 MPa, with a dry density of 850 kg/m^3^, a water resistance of 0.70, and a thermal conductivity of 0.19 W/m⋅K [25]. Nevertheless, further research is necessary to examine the influence of sediments on the physico-chemical properties of foamed concrete and its long-term stability, as there is limited literature available on this topic.

## 7. Summary and Discussion

The transformation of polluted sediments into highly valuable construction materials offers significant economic and environmental benefits. For ex situ treatments, OPC-based binders have been most widely used in solidification/stabilization technology for addressing the issues of heavy metal contaminants. However, the production process of cement emits more greenhouse gases and greater energy consumption. It is important to note that different treatment technologies have their strengths and weaknesses, and the choice should be based on sediment properties [86,87]. Incorporating waste resources with OPC as binders for solidification/stabilization treatment in immobilizing heavy metal contaminants not only aids in reducing CO_2_ emissions but also supports sustainable environmental developments. This approach addresses the problem of mitigating global warming and reduces the need for landfill disposal by reusing waste from industries to remediate heavy metal contamination, followed by the production of valuable artifacts.

The addition of OPC-based binders can increase the pH of mixtures, and the subsequent leachability of metal ions can be limited through precipitation. But, the calcium silicate hydrate (C-S-H), calcium aluminum silicate hydrate (C-A-S-H), and ettringite generated during the pozzolanic reaction can also immobilize ions of heavy metals through adsorption on the surface, ion exchanges, and encapsulation.

Soda residue (SR) and red mud (RM) are suitable for heavy metal immobilization through precipitation in an alkaline environment. But, granulated blast furnace slag (GGBS), pulverized fly ash (PFA), metakaolin, and incinerated sewage sludge ash (ISSA) have a low pH and can be used to immobilize heavy metals by blending them in high pH media.

Through different treatment techniques, sediments, despite being geomaterials of high complexity [3,88], have been successfully transformed into sustainable construction materials such as supplementary cementitious materials (SCMs), fill materials, paving blocks, partition blocks, and ready-mixed and foamed concretes. The presence of excessive fines could potentially decrease the compressive strength with a weakening of the granular structures. However, for sediment-derived products as non-load-bearing components, a lower compressive strength may be sufficient, and alternative binders to OPC can be considered. It is essential to carry out further research on the long-term stability and environmental impacts of sediment-derived construction materials for large-scale applications. The reuse of sediments can be a valuable alternative, provided that an appropriate mix design and curing conditions are employed to enhance the geotechnical properties of sediments and to meet end-of-waste criteria. 

Moreover, the identification of the remediation technology to be adopted is strictly related to the characteristics of the sediment. Assessing the most appropriate remediation technology using a multi-criteria approach can provide a strong and acceptable framework. However, conducting a broader analysis, especially based on life-cycle assessments, can aid in determining the most suitable remediation solutions. The factors affecting effectiveness include (a) the concentration of heavy metals, (b) the proportion of OPC-based binders, (c) the pH, (d) the chemical compounds and their sediment texture, (e) the moisture content, and (f) the curing time. 

## Figures and Tables

**Table 2:** The recent research experiments of OPC-based binders in heavy metal immobilization.

Characterization	Heavy Metals to Be Stabilized (Initial Concentration)	Binding Materials and Proportion	Curing Condition	Concluding Remarks	References
pH = 8.75; clay, 37.5 wt%; silt, 43.0 wt%; sand, 19.4 wt%; moisture content, 44.9%; organic matter on dry solids, 14.4 wt%; salinity, 3.41 mS/cm conductivity.	Metals; PAHs; PCBs	Lime with additives activated carbon and organoclay; water-to-solid ratio, 0.7; total reagents, 10–15%.	Samples were kept at 20 °C and an 80% moisture content in the curing phase for 28 and 56 days.	The compressive strength of sediment blocks is 28.1 kPa; leaching values are below the limit target at 56 days.	De Gisi et al. (2020) [4]
-	As (6.3 ppm)	Lime dust, 5–15 wt%; cement kiln dust (CKD), 15–30 wt%.	Cured for 28 days, and temperature not specified.	Unconfined compressive strength: 0.245 to 0.761 MPa at 28 days; leaching concentration, 0.0222–0.999 ppm.	Eisa et al. (2020) [6]
Water content, 22.54%; sp. gravity, 2.65; LL, 39.9%; PL, 22.2%; PI, 17.7%; density, 1.95 g/cm^3^.	Pb (1000 and 5000 mg/kg)	Fly ash and soda residue; FA/soil ratio, 0.05, 0.1, and 0.15; SR/soil ratio, 0.1, 0.2 and 0.3.	Humidity, 95 ± 5% and a temperature of 20 °C; 0, 3, 7, and 28 days.	Maximum UCS, 1.1 MPa for FA/soil 0.15 and 2.5 MPa for SR/soil 0.3 at 28 days; the leaching concentration of 1000 mg/kg samples was lower than the limit (<5 mg/L), but others were above the limit.	Liu et al. (2018) [13]
pH = 9.10; clay, 40.25 wt%; silt, 45.49 wt%; sand, 14.26 wt%; moisture content, 53.33%; organic matter on dry solids, 20.00%.	Metals (As, Co, Pb, Ni, Cu, Cr, and Zn); PAHs; PCBs	Cement, 15, 7.5, and 0 wt%; lime, 0, 7.5, and 15 wt%; total binder, 15 wt%; water-to-solid ratio = 1.	The samples were kept at 20 ± 5 °C and 80% moisture content for 7, 14, and 28 days.	The mobility of metals was influenced by pH and curing time. The presence of organic contamination affects the hydration of binders and, subsequently, metal immobilization and the final hardening process. Leaching: at 28 days, all values were below the target limits, except Cu and Ni.	Todaro et al. (2020) [15]
pH= 5.2–6.7; sand, 5.4–47.8 wt%; silt, 46.3–62.3 wt%; clay, 5.9–32.3 wt%; density, 1.38–1.66 g/cm^3^; organic matter, 0.25–1.26 wt%.	As (>400 mg/kg)	Cement, 80 wt% and 50 wt%; fuel ash (FA), furnace bottom ash (FBA), and ground granulated blast furnace slag (GGBS), 20 wt% and 50 wt%, respectively; w/s ratio, 0.3–0.5.	Cured at 60 ± 2 °C for 7 days.	Partial OPC replacement by FA, GGBS, and FBA showed a corresponding decline in the compressive strength and leaching concentration (0.02–0.01 mg/L).	Li et al. (2017) [20]
Water content, 20.18%; sp. gravity, 2.72; LL, 49.6%; PL, 23.10%; PI, 18.60%; density, 1.92 g/cm^3^.	Zn (1000, 5000, And 10,000 mg/kg)	Cement, 50 wt%; soda residue, 50 wt%.	Cured at 20 °C for 28 days.	The acidic environment made hydrogen ions react with the internal substances of filled soil products, with a maximum UCS of 2.66, 2.34, and 2.03 MPa at 28 days for Zn^2+^ concentrations of 0.1%, 0.5%, and 1.0%, respectively; leaching concentration, 0.233–0.642 mg/L.	Zha et al. (2021) [23]
pH = 7.34; sand, 13 wt%; silt, 62 wt%; clay, 25 wt%.	Pb (500, 1000, and 5000 mg/kg)	OPC and incinerated sewage sludge ash (ISSA) in the proportion of 1:0, 4:1, and 1:1; w/b ratio 0.5.	Cured at 23 ± 2 °C and 90% humidity for 7 and 28 days.	After 28 days, the maximum UCS was 8.5, 7.5, and 10 MPa with OPC only; and 1.8, 1.5, and 2.5 MPa for OPC:ISSA (1:0, 4:1, and 1:1); the leaching concentration was reduced to <1 mg/L.	Li et al. (2017) [27]
-	Cu, Pb, Cd, and Zn	Fly ash and silica fume ratio FA:SF, 1.0:0 to 0.80:0.20; water-to-solid ratio, 0.4.	Under environmental conditions.	Compressive strength was 5.4 MPa for the sample with only fly ash and 22.3 MPa; the leaching concentrations of Cu and Zn were 0.02 and 0.03 mg/L, respectively, but Pb and Cd were converted into a stable state.	Li et al. (2014) [28]
River sand, < 2 mm, gravels, 5–20 mm; metakaolin size, 0.1–27.5 µm and d_50_ = 4.8 µm; fly ash particle size, 0.1–207.5 µm and d_50_ = 17.3 µm.	-	Fly ash and metakaolin by mixing with a ratio of 1:1 and using potassium silicate excitation, 40 wt%; water-to-binder ratio, 0.35, 0.4, and 0.45.	Watered and cured under natural environmental conditions for 3 and 7 days.	The maximum compressive strengths with the highest content of metakaolin and fly ash were 46.6 MPa and 68 MPa, respectively, at 3 and 7 days of curing.	Xing et al. (2020) [31]
pH = 8.51; sand, 44.64 wt%; silt, 16.69 wt%; clay, 0.34 wt%; gravel, 38.33 wt%; water content, 77.71%; salinity, 24.8 g/kg; organic matter, 4.2%; sp. gravity, 2.49.	Cu and Zn (Cu, 74.04 mg/kg; Zn, 154.72 mg/kg)	Sewage sludge ash (ISSA) at 0.2 mass ratio and cement/lime at 0.05/0.1 mass ratios.	Cured at 23 ± 2 °C for 3, 7, 14, 28, and 90 days.	The maximum UCS with cement and ISSA was 4.6 MPa, and with lime and ISSA, it was 2.5 MPa at 90 days; Cu, 0.075–0.3 mg/L; Zn, 0.01–4.00 mg/L.	Li et al. (2021) [33]
Dry density, 1.92 g/cm3; sp. gravity, 2.67; LL, 49.4%; PL, 24.8%; PI, 25.6; LI, 0.03; water content, 25.5%.	Pb, Zn, and Cr (10,000 mg/kg)	Cement. 25 wt%; fly ash. 75 wt%.	Cured at 22 ± 1 °C for 0, 7, 28, 90 days.	The maximum UCS was 2.5 MPa at 28 days; Pb, 15–20 mg/L; Zn, 10–15 mg/L; Cr, 0.15–0.3 mg/L.	Zha et al. (2019) [53]
Calcined shell pH, up to 10.7; electrical conductivities, 1.19–3.55 dS/m.	As, Cr, and Hg	Calcined MS, sewage sludge. and wood ash; solid-to-water ratio, 1:2.5.	-	The adsorption of As(V) and Hg(II) were higher (>88%) and low for Cr(VI) (up to 30%).	Sceo et al. (2013) [45]
-	Pb, Zn, and Cu (2500, 5000, and 10,000 mg/kg)	Cement, 10–100 wt%; red mud, 40–100 wt%; phosphogypsum, 10–70 wt%; fly ash, 30–70 wt%.	Cured at 20 ± 0.5 °C for 7, 14, and 28 days.	The addition of phosphogypsum significantly increases the strength of the soil products, and the main mechanism of stabilizing HMs is the adsorption of red mud and ettringite and ion exchanges by Ca or Al in ettringite; Cu, 1.5 mg/L; Pb, 1 mg/L; Zn, 3 mg/L (minimum).	Wang et al. (2020) [58]
pH 7.2; salinity, 35.9 g/kg; organic matter, 6.9%; gravel, 27 wt%; sand, 58 wt%; silt and clay, 15 wt%.	As (201 mg/kg)	Cement, 80 wt%; red mud (RM), blast furnace slag (BS), and metakaolin (MK), 20 wt%; sediment-to-binder ratio, 7:3 and 9:1 for in situ and ex situ.	Cured at 23 ± 1 °C for 7 and 28 days.	The RM-incorporated binder can be used for low-cost and low-carbon S/S treatments; the MK-incorporated binder is superior in mechanical strength (at 9.0 MPa in situ); leaching concentration, 0.1–0.5 mg/L.	Wang et al. (2019) [64]
